# *Tsc2* mutation rather than *Tsc1* mutation dominantly causes a social deficit in a mouse model of tuberous sclerosis complex

**DOI:** 10.1186/s40246-023-00450-2

**Published:** 2023-02-02

**Authors:** Hirofumi Kashii, Shinya Kasai, Atsushi Sato, Yoko Hagino, Yasumasa Nishito, Toshiyuki Kobayashi, Okio Hino, Masashi Mizuguchi, Kazutaka Ikeda

**Affiliations:** 1 Addictive Substance Project, Tokyo Metropolitan Institute of Medical Science, 2-1-6 Kamikitazawa, Setagaya-Ku, Tokyo, 156-8506 Japan; 2 Department of Neuropediatrics, Tokyo Metropolitan Neurological Hospital, 2-6-1 Musashidai, Fuchu, Tokyo, 183-0042 Japan; 3 Department of Pediatrics, The University of Tokyo Hospital, 7-3-1 Hongo, Bunkyo-Ku, Tokyo, 113-8655 Japan; 4 Center for Basic Technology Research, Tokyo Metropolitan Institute of Medical Science, 2-1-6 Kamikitazawa, Setagaya-Ku, Tokyo, 156-8506 Japan; 5 Department of Pathology and Oncology, Juntendo University School of Medicine, 2-1-1 Hongo, Bunkyo-Ku, Tokyo, 113-8421 Japan; 6 Department of Pediatrics, National Rehabilitation Center for Children with Disabilities, 1-1-10 Komone, Itabashi-Ku, Tokyo, 173-0037 Japan

**Keywords:** Double mutations, *Pdlim2*, Rapamycin, *Tsc1*, *Tsc2*, Tuberous sclerosis complex

## Abstract

**Background:**

Tuberous sclerosis complex (TSC) is an autosomal dominant disorder that is associated with neurological symptoms, including autism spectrum disorder. Tuberous sclerosis complex is caused by pathogenic germline mutations of either the *TSC1* or *TSC2* gene, but somatic mutations were identified in both genes, and the combined effects of *TSC1* and *TSC2* mutations have been unknown.

**Methods:**

The present study investigated social behaviors by the social interaction test and three-chambered sociability tests, effects of rapamycin treatment, and gene expression profiles with a gene expression microarray in *Tsc1* and *Tsc2* double heterozygous mutant (*TscD*^+/−^) mice.

**Results:**

*TscD*^+/−^ mice exhibited impairments in social behaviors, and the severity of impairments was similar to *Tsc2*^+/−^ mice rather than *Tsc1*^+/−^ mice. Impairments in social behaviors were rescued by rapamycin treatment in all mutant mice. Gene expression profiles in the brain were greatly altered in *TscD*^+/−^ mice more than in *Tsc1*^+/−^ and *Tsc2*^+/−^ mice. The gene expression changes compared with wild type (WT) mice were similar between *TscD*^+/−^ and *Tsc2*^+/−^ mice, and the overlapping genes whose expression was altered in mutant mice compared with WT mice were enriched in the neoplasm- and inflammation-related canonical pathways. The “signal transducer and activator of transcription 3, interferon regulatory factor 1, interferon regulatory factor 4, interleukin-2R α chain, and interferon-γ” signaling pathway, which is initiated from signal transducer and activator of transcription 4 and PDZ and LIM domain protein 2, was associated with impairments in social behaviors in all mutant mice.

**Limitations:**

It is unclear whether the signaling pathway also plays a critical role in autism spectrum disorders not caused by *Tsc1* and *Tsc2* mutations.

**Conclusions:**

These findings suggest that *TSC1* and *TSC2* double mutations cause autistic behaviors similarly to *TSC2* mutations, although significant changes in gene expression were attributable to the double mutations. These findings contribute to the knowledge of genotype–phenotype correlations in TSC and suggest that mutations in both the *TSC1* and *TSC2* genes act in concert to cause neurological symptoms, including autism spectrum disorder.

**Supplementary Information:**

The online version contains supplementary material available at 10.1186/s40246-023-00450-2.

## Background

Autism spectrum disorder (ASD) is a complex neurodevelopmental disorder that is characterized by two core behavioral symptoms: deficits in social communication and social interaction, and restricted, repetitive patterns of behaviors, interests, or activities [1]. Despite its high clinical and etiological heterogeneity, recent genetic studies have revealed that ASD appears to share neurobiological mechanisms and be attributable to the dysregulation of some convergent pathways [2–5]. One such pathway is the mechanistic target of rapamycin complex 1 (mTORC1) signaling cascade [6–9].

Tuberous sclerosis complex (TSC), a prototypical mTORopathy, is an autosomal dominant disorder. Up to 50% of patients with TSC have comorbid ASD. The prevalence of TSC in overall ASD was estimated to be 0.01 [10–12]. Thus, TSC is one of the most common diseases that is responsible for syndromic ASD. Tuberous sclerosis complex is considered to be caused by pathogenic mutations in the TSC complex subunit 1 (*TSC1*) or *TSC2* gene and is unique in the similarity of clinical phenotypes between pathogenic mutations in the two genes [13–15]. TSC1 and TSC2 proteins form a complex with complemental TBC domain family member 7, which suppresses a small guanosine triphosphate-binding protein, Rheb, and negatively regulates the mTORC1 signaling cascade [16]. The excessive activation of mTORC1 signaling that is caused by pathogenic mutations in the two genes is assumed to be the primary pathophysiological mechanism of TSC [17]. According to Knudson’s two-hit theory, TSC-associated tumors develop because of biallelic inactivation of the *TSC1* or *TSC2* gene [18]. Indeed, the loss of heterozygosity (LOH) of either gene is often detected in most TSC-associated tumors, and more than half of brain cortical tubers lack LOH [19–21], suggesting that the heterozygosity of *TSC1* or *TSC2* is sufficient to cause neuronal abnormalities [22].

Genotype–phenotype correlation studies suggest that *TSC2* pathogenic mutations rather than *TSC1* pathogenic mutations are associated with severe phenotypic manifestations, including early-onset seizures, a high rate of complications that are associated with ASD, low cognitive function, and a high number of renal angiomyolipomas [15, 23–26]. Somatic *TSC1* mutations were identified in lesions from patients with a pathogenic germline *TSC2* mutation, suggesting that *TSC1*/*TSC2* double heterozygous mutations may promote tumorigenesis [21, 27, 28]. Therefore, evaluations of manifestations of double heterozygous pathogenic mutations compared with mutations of either gene alone will provide insights into the pathophysiology of TSC. However, previous studies exclusively focused on pathogenic mutations of either gene alone, and few studies have described *TSC1*/*TSC2* double mutations [29–31].

The homozygous loss of either *Tsc1* or *Tsc2* in mice causes embryonic lethality [32, 33]. Conventional heterozygous mice recapitulate some neuropsychiatric phenotypes of TSC in humans, including learning and memory deficits and social impairments without apparent brain lesions [34–36]. Additionally, transient treatment with an mTORC1 inhibitor successfully rescued these deficits.

The present study focused on the impact of *Tsc1* and *Tsc2* double heterozygous mutations in mouse models. We analyzed social behaviors and gene expression profiles in *Tsc1* and *Tsc2* double heterozygous mice and assessed the therapeutic effects of the mTOR inhibitor rapamycin on autistic-like behaviors.

## Materials and methods

### Mice

*Tsc1* heterozygous knockout (*Tsc1*^+/−^) and *Tsc2* heterozygous knockout (*Tsc2*^+/−^) mice were generated and maintained on a C57BL6/J background as previously described [32, 33]. A *Tsc1*^+/−^ mouse and a *Tsc2*^+/−^ mouse were crossed to produce *Tsc1*^+/+^/*Tsc2*^+/+^ (WT) mice, *Tsc1*^+/−^/*Tsc2*^+/+^ (*Tsc1*^+/−^) mice, *Tsc1*^+/+^/*Tsc2*^+/−^ (*Tsc2*^+/−^) mice, and *Tsc1*^+/−^/*Tsc2*^+/−^ (*TscD*^+/−^) mice. C57BL/6J mice were purchased from CLEA Japan (Tokyo, Japan). After weaning at 3 weeks of age, all mice were maintained separately by sex (two to five mice per cage) in ventilated racks at 22 °C ± 2 °C under a 12 h/12 h light/dark cycle (lights on 8:00 A.M. to 8:00 P.M.) with access to a standard laboratory diet and water ad libitum.

### Drug

Rapamycin (LC Laboratories, Woburn, MA, USA) was dissolved to 0.5 mg ml^−1^ with 10% dimethylsulfoxide (vehicle) and administered intraperitoneally in a volume of 10 ml kg^−1^ once daily for 2 days. The behavioral tests and brain collection for gene expression analysis began 24 h after the second rapamycin administration.

### Behavioral tests

The hole board test, self-grooming test, social interaction test, three-chambered sociability test, elevated plus maze test, and social transmission of food preference test were performed with male and female mice at 14.4–21.6, 44.4–46.9, 17.1–43.4, 15.6–43.4, 15.6–26.1, and 16.0–21.7 weeks of age between 10:00 A.M. and 4:00 P.M., respectively. The number of WT, *Tsc1*^+/−^, *Tsc2*^+/−^, and *TscD*^+/−^ mice that were used in the behavioral tests were 13–19, 14–20, 15–20, and 14–22, respectively.

*Hole board test* Each mouse was placed in a hole board apparatus (500 mm × 500 mm × 400 mm; Muromachi Kikai, Tokyo, Japan) with four holes (38 mm diameter) and allowed to freely explore it for 30 min. Behavior was automatically analyzed for counts of head dipping using a video tracking system.

*Self-grooming test* Each mouse was individually placed in a new cage without bedding material. After an initial 10-min habituation period, the animal was video-recorded for 10 min. The number of episodes of self-grooming and rearing behavior was measured.

*Social interaction test* Each mouse was left alone in its home cage for 15 min. A novel mouse (C57BL/6J) of the same sex and size was then introduced to the cage. The behavior of the test mouse was video recorded for 10 min. The total duration of active interaction (anogenital sniffing, allogrooming, close following, and mounting) was measured.

*Three-chambered sociability tests* A three-chamber arena (500 mm × 500 mm × 400 mm) was used to evaluate social approach, preference for social novelty, and social preference. After a 10 min habituation period, an unfamiliar C57BL/6J mouse of the same sex (stranger 1) was introduced to the columnar wire cage (100 mm diameter, 100 mm height) in a side chamber, and a metal block (object) was placed in another cage on the other side chamber. The test mouse was allowed to explore the entire arena for 10 min (Session 1: social approach). The block was then replaced with another unfamiliar mouse (stranger 2), and the test mouse was examined for an additional 10 min (Session 2: social novelty). The test mouse was then presented with another unfamiliar mouse (stranger 3) and a cagemate of the test mouse for 10 min (Session 3: social preference). The amount of time spent around each cage was automatically measured using a video tracking system. The approach-avoidance score was calculated as the following: *(time exploring stranger 3)* − *(time exploring cagemate)*.

*Elevated plus maze test* Each mouse was individually placed in the center of the elevated plus maze apparatus (Muromachi Kikai) and allowed to freely explore the apparatus for 10 min. Total distance traveled and time spent on the open arms were automatically measured using a DV-Track Video Tracking System (Muromachi Kikai).

*Social transmission of food preference test* Twenty-four hours before testing, the test mouse was placed in a novel cage with a specially designed food jar that was filled with powdered chow. Eighteen hours before testing, the feeding jars were removed, and the mice were deprived of food until testing. To begin testing, a “demonstrator” mouse was given a food jar that was filled with powdered chow that was flavored by mixing it with either 1% ground cinnamon or 2% powdered cocoa. After 1 h, the food jar was removed and weighed to ensure that at least 0.2 g of food had been consumed. The demonstrator mouse was then immediately placed in the cage of the “observer” test mouse, and interactions between the demonstrator mouse and observer mouse were allowed for 30 min. The demonstrator mouse was then removed, and two jars of powdered food (one of each flavor) were placed in the cage with the observer mouse for 1 h. At the end of the 1 h choice session, the food jars were removed and weighed. The demonstrator mice were C57BL/6J mice that were matched to the test observer mouse according to weight and sex.

### Whole transcriptome analysis

Total RNA was isolated from the right brain hemisphere of 12-week-old male mice using Sepasol-RNA I Super G (nacalai tesque, Kyoto, Japan) and purified with RNeasy Mini (Qiagen, Tokyo, Japan). Only RNA with an RNA Integrity Number above 8.0, determined by an Agilent Bioanalyzer (Agilent Technologies, Tokyo, Japan), was used for gene expression analysis. Cy3-labeled cRNA was prepared using the Low Input Quick Amp Labeling Kit (Agilent Technologies) according to the manufacturer’s protocol, hybridized to the SurePrint G3 Mouse Gene Expression v2 8 × 60 K Microarray (Agilent Technologies), and scanned using a SureScan Microarray Scanner (Agilent Technologies).

Microarray data were measured using Feature Extraction software (Agilent Technologies) and analyzed using GeneSpring GX software (Agilent Technologies). Differentially expressed transcripts (DETs) were identified by two-way analysis of variance (ANOVA) with *p* < 0.05. Common transcripts between more than two DETs were selected instead of multiple testing correction to minimize false positives. Clustering analysis was performed using hierarchical clustering analysis with the default Euclidean distance metric and default Ward’s linkage rule.

### Gene ontology and pathway enrichment analyses

Gene ontology (GO) and pathway enrichment analyses were performed using MetaCore version 6.31 (https://portal.genego.com/cgi/data_manager.cgi; accessed July 22, 2022). Map Folders and GO Process Networks are mainly based on biochemical and signaling cascades and cellular processes, respectively. The statistical significance value and false discovery rate (FDR) were used to rank GO. Canonical pathway modeling was used to identify pathways with statistical significance, zScore, and gScore by considering the number of objects of datasets and canonical pathways in the networks.

### Statistical analysis

The statistical analyses of behavioral data were performed using SPSS Statistics 24 software (IBM Japan Ltd., Tokyo, Japan). Motor, sensory, and social behaviors between mutant and WT mice were statistically evaluated using one-way ANOVA followed by the Tukey Honestly Significant Difference (HSD) test. Unpaired *t*-tests were used to evaluate differences between vehicle and rapamycin treatment in the social interaction test. Paired *t*-tests were used to evaluate differences between familiar and stranger mice in the three-chambered sociability tests and between cued food and non-cued food in the social transmission of food preference test. Values of *p* < 0.05 were considered statistically significant.

## Results

### Autistic-like behaviors in Tsc1^+/−^, Tsc2^+/−^, and TscD^+/−^ mice

**Fig. 1:**
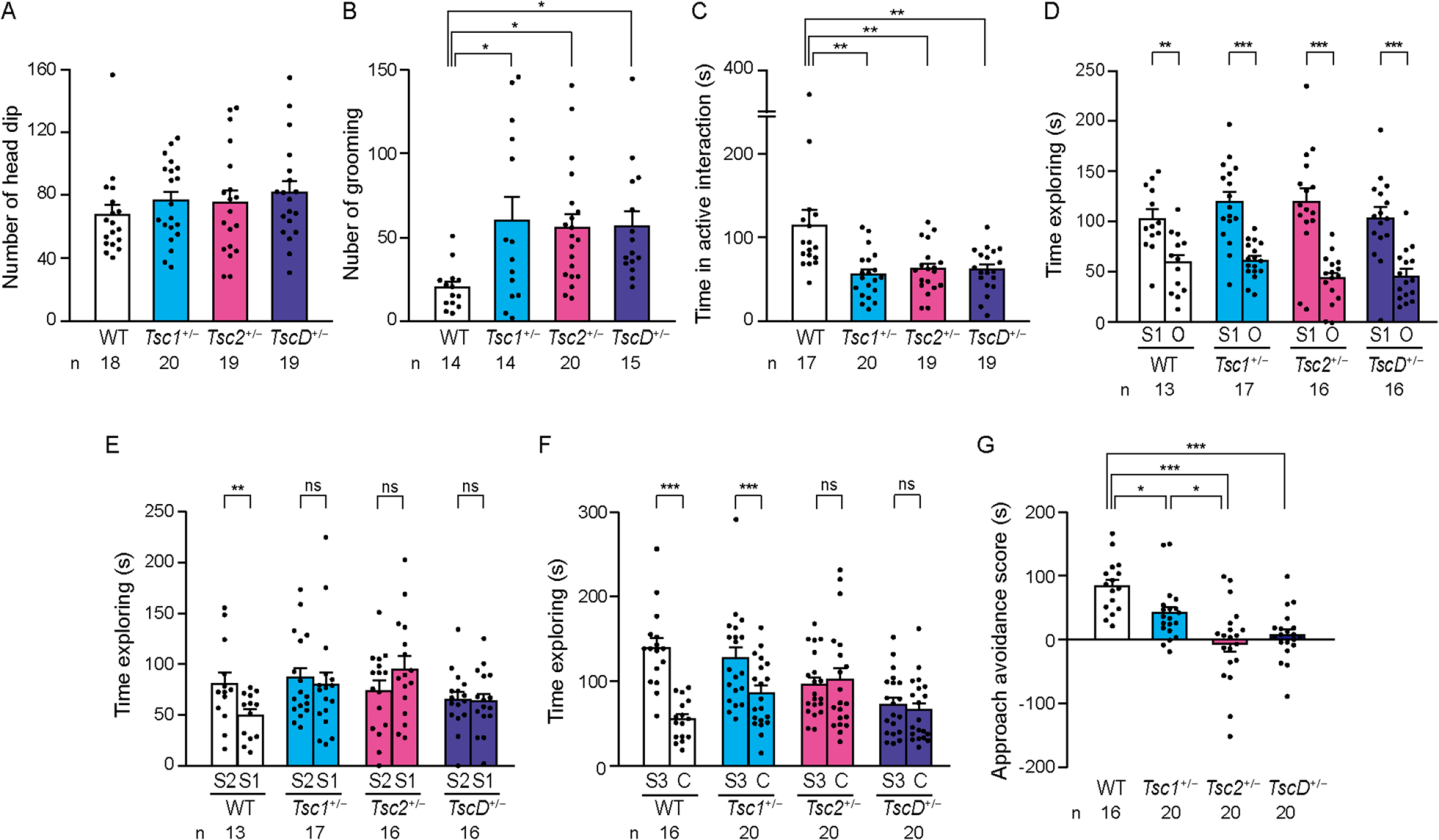
Social behaviors in *Tsc1*^+/−^, *Tsc2*^+/−^, and *TscD*^+/−^ mice. **A–C** Frequencies of head-dipping (**A**) and self-grooming (**B**) and time spent in active interaction behaviors (**C**) in *Tsc1*^+/−^, *Tsc2*^+/−^, and *TscD*^+/−^ mice in the hole board test, self-grooming test, and social interaction test, respectively. **D** Time spent exploring (social approach) a stranger mouse (S1) and non-social object (O) in the three-chamber test. **E** Time spent exploring a familiar mouse (S1) and stranger mouse (S2) in the social-novelty preference test. **F** Time spent exploring a cagemate (C) and stranger mouse (S3) in the social preference test. **G** Approach avoidance score calculated as the following: *(time exploring S3)* – *(time exploring C)*. *p < 0.05; **p < 0.01; ***p < 0.001, one-way ANOVA followed by Tukey HSD test (**A**–**C**, **G**) and paired *t*-test (**D–F**). Each bar indicates the mean ± SEM

The homozygous knockout of either the *Tsc1* or *Tsc2* gene causes embryonic lethality in mice, whereas both *Tsc1*^+/−^ and *Tsc2*^+/−^ mice are apparently healthy, do not die prematurely, and lack apparent cerebral lesions and spontaneous seizures [32, 33]. *TscD*^+/−^ mice were born at the expected Mendelian frequency and exhibited no apparent spontaneous seizures, no early death, and no delay of physical development (data not shown). Adult *Tsc1*^+/−^ and *Tsc2*^+/−^ mice have been reported to exhibit normal motor and sensory behaviors [36]. Similar to *Tsc1*^+/−^ and *Tsc2*^+/−^ mice, *TscD*^+/−^ mice did not display any significant differences in motor or sensory behaviors in the hole board test (Fig. 1A), elevated plus maze test, or self-grooming test compared with WT mice (Additional file 1: Fig. S1A–C). *TscD*^+/−^ mice normally discriminated the scent of a food that was presented by stranger mice in the social transmission of food preference test, similar to *Tsc1*^+/−^ and *Tsc2*^+/−^ mice, indicating that these mutant mice can differentiate novel nonsocial stimuli that are transmitted through social interaction (Additional file 1: Fig. S1D). *TscD*^+/−^ mice exhibited more frequent self-grooming behavior than WT mice, similar to *Tsc1*^+/−^ and *Tsc2*^+/−^ mice (*p* = 0.024, 0.031, and 0.042 for *Tsc1*^+/−^, *Tsc2*^+/−^, and *TscD*^+/−^ mice, respectively, Tukey HSD test; Fig. 1B). *TscD*^+/−^ mice also exhibited a reduction in interaction time compared with WT mice, similar to *Tsc1*^+/−^ and *Tsc2*^+/−^ mice, in the social interaction test (*p* = 0.001, 0.004, and 0.004 for *Tsc1*^+/−^, *Tsc2*^+/−^, and *TscD*^+/−^ mice, respectively, Tukey HSD test; Fig. 1C). In the social approach session of the three-chambered sociability test, *TscD*^+/−^ mice exhibited normal social preference, with a significant difference between the novel mouse and non-social object, similar to WT, *Tsc1*^+/−^, and *Tsc2*^+/−^ mice (Fig. 1D). In the social novelty session, WT mice spent significantly more time exploring the novel mouse than the familiar mouse, whereas *TscD*^+/−^ mice exhibited no significant preference for social novelty, similar to *Tsc1*^+/−^ and *Tsc2*^+/−^ mice (*p* = 0.005, 0.581, 0.099, and 0.845 for WT, *Tsc1*^+/−^, *Tsc2*^+/−^, and *TscD*^+/−^ mice, respectively, paired *t*-test; Fig. 1E). In the social preference session, *TscD*^+/−^ mice spent a similar amount of time exploring both the novel mouse and the cagemate, similar to *Tsc2*^+/−^ mice, whereas WT and *Tsc1*^+/−^ mice preferred the novel mouse significantly more than the cagemate (*p* < 0.001, *p* < 0.001, *p* = 0.725, and *p* = 0.422 for WT, *Tsc1*^+/−^, *Tsc2*^+/−^, and *TscD*^+/−^ mice, respectively, paired *t*-test; Fig. 1F). The calculated approach-avoidance score revealed a severe reduction of social preference for a novel mouse in *Tsc2*^+/−^ and *TscD*^+/−^ mice rather than *Tsc1*^+/−^ mice (*p* = 0.045, *p* < 0.001, *p* < 0.001 for *Tsc1*^+/−^, *Tsc2*^+/−^, and *TscD*^+/−^ mice, respectively, compared with WT mice; *p* = 0.015, *Tsc1*^+/−^ mice compared with *Tsc2*^+/−^ mice, Tukey HSD test; Fig. 1G).

### Rapamycin ameliorated impairments in social behaviors in TscD^+/−^ mice similarly to Tsc1^+/−^ and Tsc2^+/−^ mice

**Fig. 2:**
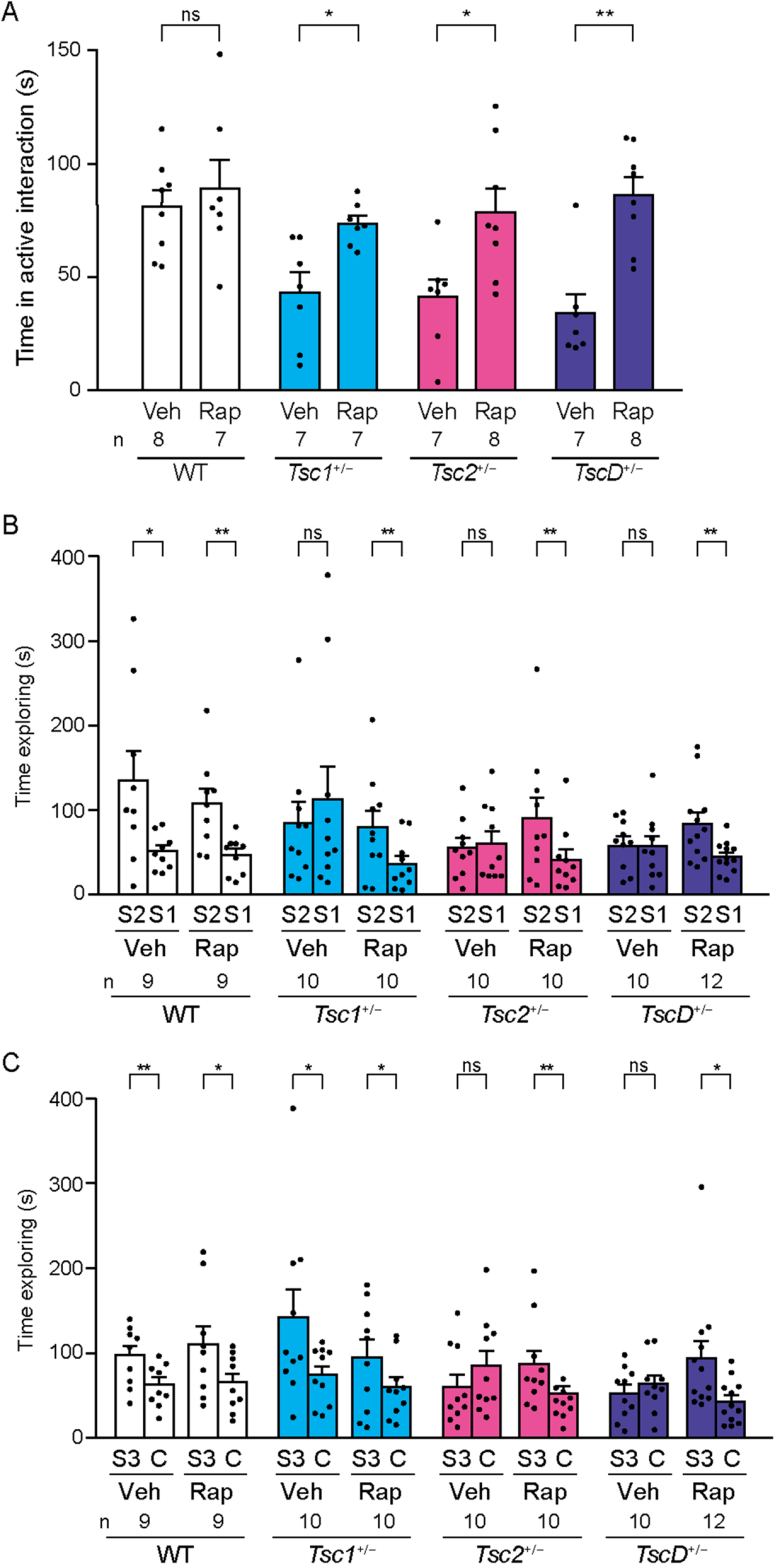
Effects of rapamycin on social behaviors in *Tsc1*^+/−^, *Tsc2*^+/−^, and *TscD*^+/−^ mice. **A–C** Effects of rapamycin and vehicle on the time spent in active interaction in the social interaction test (**A**), time spent exploring a familiar mouse (S1) and stranger mouse (S2) in the social novelty session of the three-chambered sociability test (**B**), and time spent exploring a stranger mouse (S3) and cagemate (C) in the social preference session of the three-chambered sociability test (**C**). *p < 0.05; **p < 0.01; ***p < 0.001, unpaired *t*-test (**A**) and paired *t*-test (**B, C**). Each bar indicates the mean ± SEM. *Veh* vehicle, *Rap* rapamycin

We previously reported that 5–10 mg kg^−1^ rapamycin ameliorated impairments in social interaction in *Tsc1*^+/−^ and *Tsc2*^+/−^ mice in the social interaction test [36]. The time spent engaged in active interaction was increased by 5 mg kg^−1^ rapamycin in *TscD*^+/−^ mice in the social interaction test similarly to *Tsc1*^+/−^ and *Tsc2*^+/−^ mice, with no effect in WT mice (*p* = 0.559, 0.012, 0.016, and 0.001 for WT, *Tsc1*^+/−^, *Tsc2*^+/−^, and *TscD*^+/−^ mice, respectively, unpaired *t*-test; Fig. 2A). Rapamycin also increased social novelty preference in *Tsc1*^+/−^ mice (*p* = 0.600 and 0.007 for vehicle and rapamycin treatment, respectively, paired *t*-test; Fig. 2B), *Tsc2*^+/−^ mice (*p* = 0.513 and 0.008 for vehicle and rapamycin treatment, respectively, paired *t*-test; Fig. 2B), and *TscD*^+/−^ mice (*p* = 0.749 and 0.003 for vehicle and rapamycin treatment, respectively, paired *t*-test; Fig. 2B) and social preference in the three-chambered sociability tests in *Tsc2*^+/−^ mice (*p* = 0.106 and 0.007 for vehicle and rapamycin treatment, respectively, paired *t*-test; Fig. 2C) and *TscD*^+/−^ mice (*p* = 0.314 and 0.042 for vehicle and rapamycin treatment, respectively, paired *t*-test; Fig. 2C), which were similar to *Tsc2*^+/−^ mice but not *Tsc1*^+/−^ mice.

### Changes in gene expression in Tsc1^+/−^, Tsc2^+/−^, and TscD^+/−^ mice

**Fig. 3:**
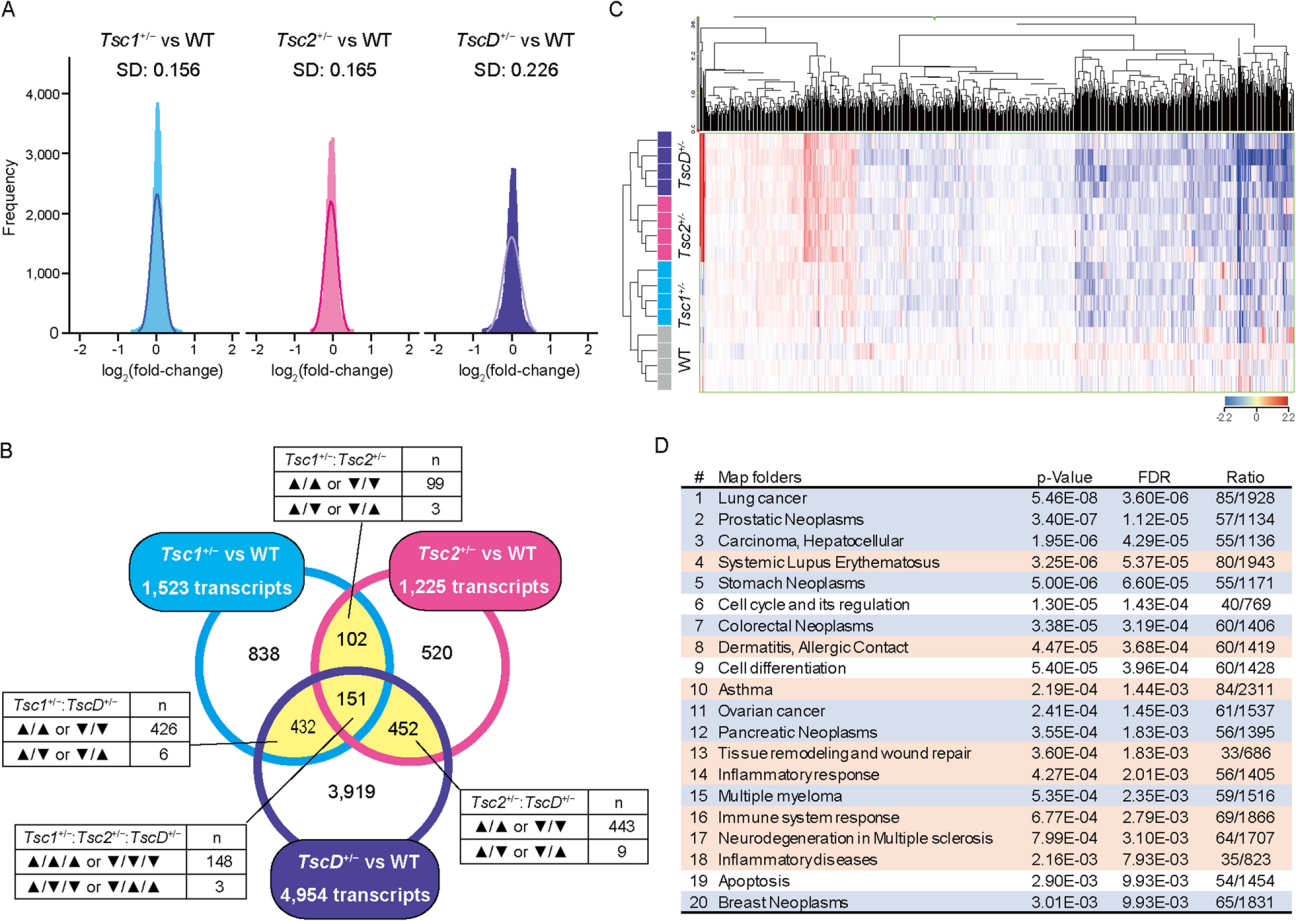
Gene expression profiles in the brain in *Tsc1*^+/−^, *Tsc2*^+/−^, and *TscD*^+/−^ mice. **A** Histograms of the log_2_ (fold-change) (*n* = 45,313, bin width = 0.02) for each mutant mouse compared with WT (cyan: *Tsc1*^+/−^ vs. WT, magenta: *Tsc2*^+/−^ vs. WT, blue: *TscD*^+/−^ vs. WT). Solid lines represent approximate normal distribution curves. *SD* standard deviation. **B** Venn diagram of three DETs between mutant and WT mice. Among overlapping transcripts (yellow area), transcripts that changed in different directions (▲: upregulated, ▼: downregulated) were excluded from each entity for further analysis. **C** Heat map of valid 1119 transcript profiles in vehicle-treated samples for four genotypes (*n* = 4 for each genotype) that were in common with at least two DETs and met the criteria. Hierarchical clustering was based on the relative expression value after baseline correction to the median of vehicle-treated WT samples (red: upregulated, blue: downregulated). **D** Top 20 rank map folders enriched for differentially expressed 1119 transcripts in mutant mice. The blue and red folders indicate cancer/neoplasm and immunity/inflammation, respectively. *FDR* false discovery rate

To evaluate similarities and differences in autistic-like behaviors in mutant mice, gene expression levels were profiled in the brain in mutant mice. After excluding control and low-intensity transcripts from a total of 56,745 transcripts that were included in the microarray, 45,313 transcripts were analyzed. The standard deviations of log_2_ (fold-change) were 0.156, 0.165, and 0.226 for *Tsc1*^+/−^, *Tsc2*^+/−^, and *TscD*^+/−^ mice, respectively, compared with WT mice, and the approximate curve showed a larger variation of log_2_ (fold-change) in *TscD*^+/−^ mice than in *Tsc1*^+/−^ and *Tsc2*^+/−^ mice (Fig. 3A).

In *Tsc1*^+/−^, *Tsc2*^+/−^, and *TscD*^+/−^ mice compared with WT mice, 1523 transcripts, 1225 transcripts, and 4954 transcripts, respectively, were identified as DETs (two-way ANOVA, *p* < 0.05; Fig. 3B). To minimize false-positive changes in gene expression, only transcripts that were in common with at least two DETs (yellow area) were the focus of the subsequent analysis. Of the 1119 transcripts that were obtained after excluding transcripts that were regulated adversely between DETs, 289 and 830 were expressed upward and downward, respectively. Hierarchical clustering analysis showed that the expression patterns of these significant transcripts in *TscD*^+/−^ mice were similar to *Tsc2*^+/−^ mice but not *Tsc1*^+/−^ mice (Fig. 3C).

Among the top 20 canonical processes that were identified in Map Folders, two major categories were included: cancer or neoplasms (e.g., lung cancer, prostatic neoplasm, hepatocellular carcinoma, and stomach neoplasm; nine processes) and diseases or pathways related to immunity or inflammation (e.g., systemic lupus erythematosus, allergic dermatitis, and asthma; eight processes; Fig. 3D). This result was consistent with the *Tsc1* and *Tsc2* gene functions of suppressing tumors and regulating immune and inflammatory responses via the mTOR pathway. Among GO process networks, the top three enrichment processes included “cellular component organization or biogenesis,” “regulation of cation channel activity,” and “nervous system development” (Additional file 3: Table S1). Furthermore, the 148 transcripts that overlapped between all three DETs were enriched in the seven significant networks in the canonical pathway modeling analysis in MetaCore (*p* < 0.05, zScore > 100; Additional file 4: Table S2).

### Differentially expressed transcripts that were reversed by rapamycin treatment in the “STAT3, IRF1, IRF4, IL-2R alpha chain, IFN-γ” pathway initiated from PDLIM2

**Fig. 4:**
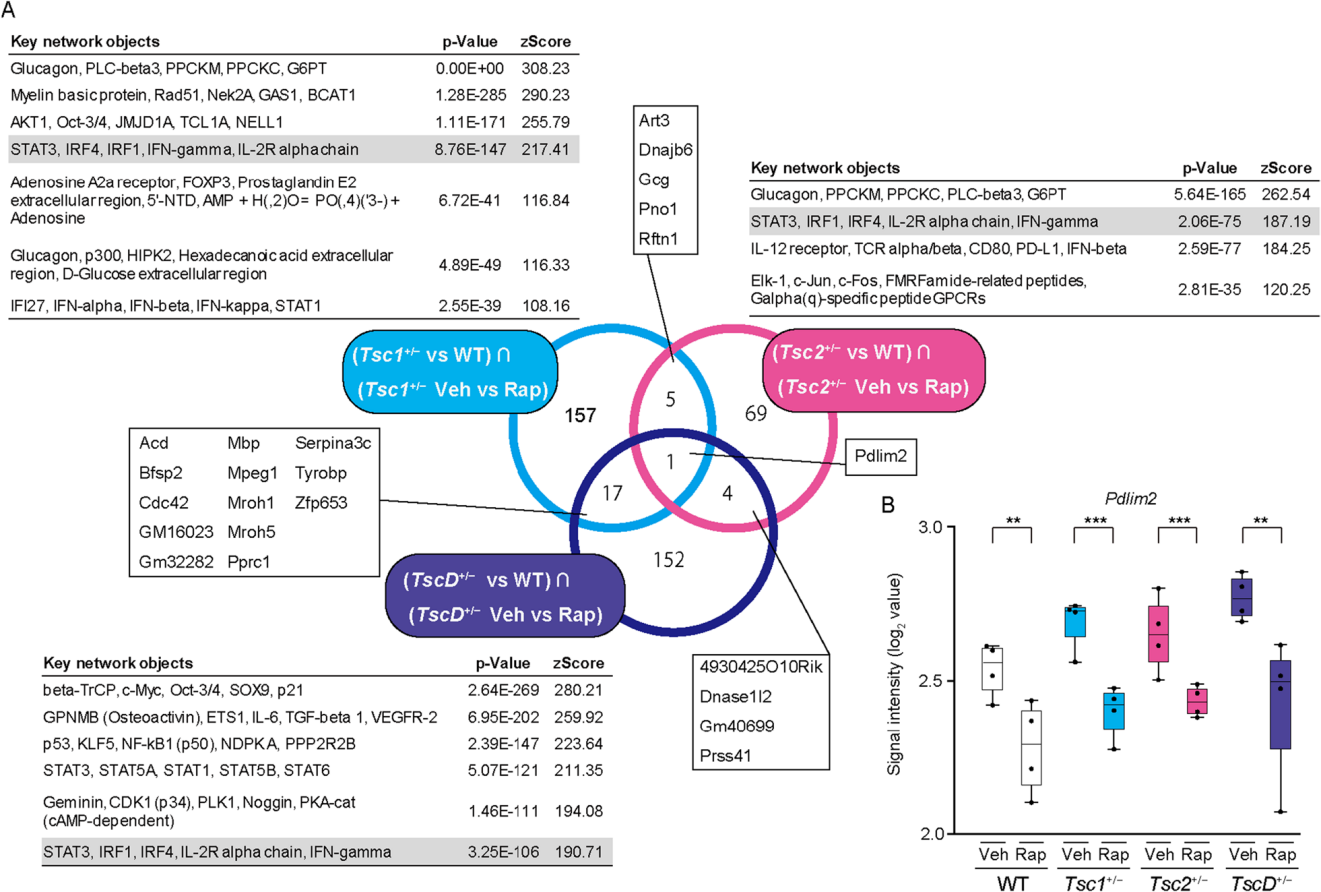
Rapamycin-induced changes in gene expression in the brain in *Tsc1*^+/−^, *Tsc2*^+/−^, and *TscD*^+/−^ mice. **A** The cyan-colored circle indicates transcripts whose expression significantly changed in *Tsc1*^+/−^ mice and was reversed by treatment with rapamycin ([*Tsc1*^+/−^ vs. WT] ∩ [*Tsc1*^+/−^ Veh vs. Rap]). Magenta- and blue-colored circles indicate gene expression changes in *Tsc2*^+/−^ and *TscD*^+/−^ mice, respectively. Canonical pathway modeling analysis for these three groups of transcripts identified seven, five, and six significant networks in *Tsc1*^+/−^, *Tsc2*^+/−^, and *TscD*^+/−^ mice, respectively (*p* < 0.05, zScore > 100). **B** Box-and-whisker plot of normalized log_2_ signal intensity for the gene expression of *Pdlim2* in vehicle- or rapamycin-treated mutant and WT mice. **p < 0.01; ***p < 0.001 (two-way ANOVA). *Veh* vehicle, *Rap* rapamycin

**Fig. 5:**
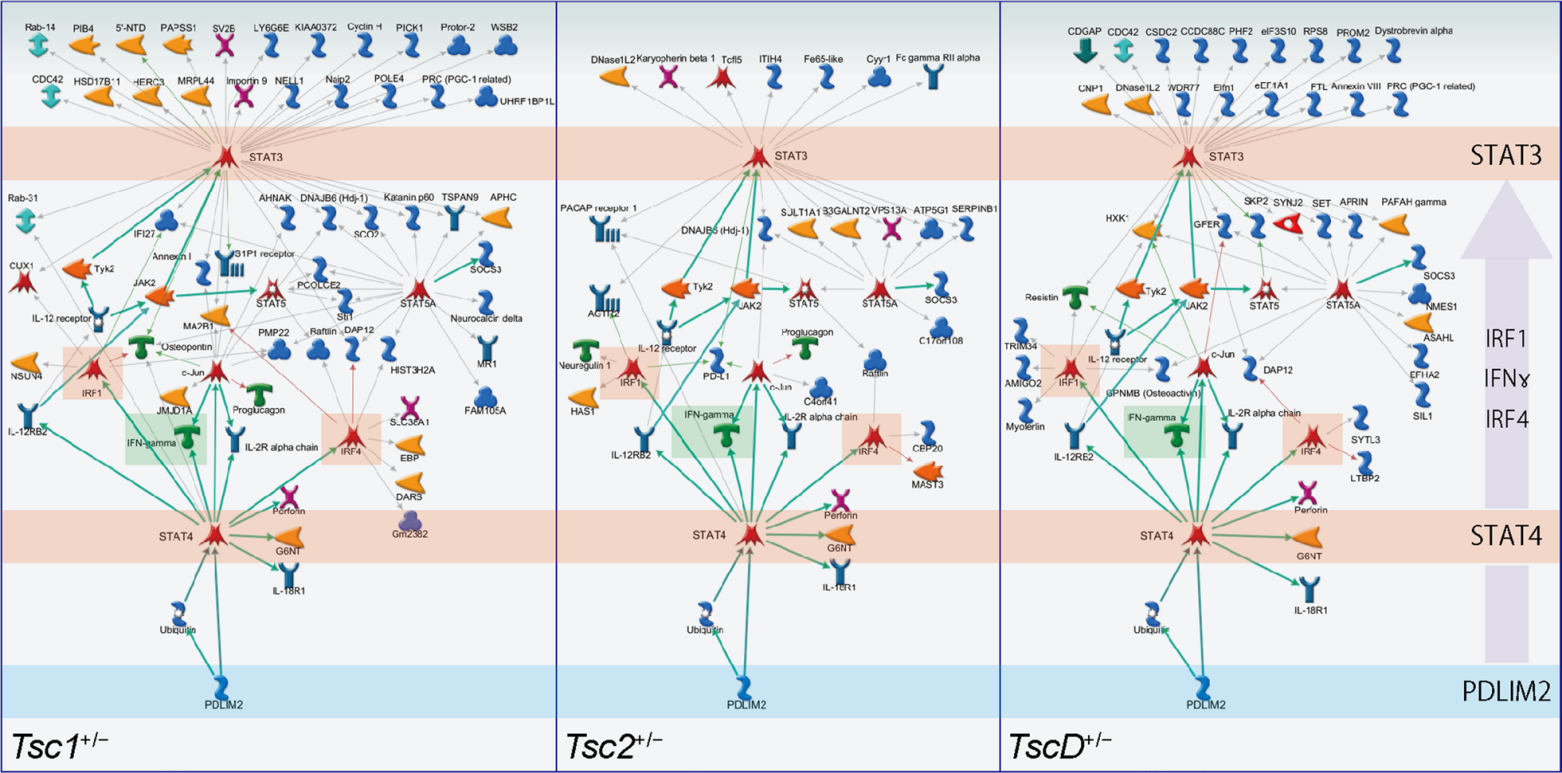
Signaling pathway associated with DETs in *Tsc1*^+/−^, *Tsc2*^+/−^, and *TscD*^+/−^ mice with rapamycin treatment. Arrows indicate directional edges, with main actions shown in green. All pathways are commonly initiated from PDLIM2 to STAT3 via STAT4, IRF1, IFNγ, and IRF in *Tsc1*^+/−^, *Tsc2*^+/−^, and *TscD*^+/−^ mice (symbol definitions can be found at https://portal.genego.com/legends/MetaCoreQuickReferenceGuide.pdf; accessed July 14, 2022)

The effect of rapamycin on transcriptional changes was investigated, and we assessed whether the effects corresponded to the results of the behavioral tests in mutant mice. Among the DETs that were obtained to compare mutant and WT mice (1523 transcripts in *Tsc1*^+/−^ vs. WT, 1225 transcripts in *Tsc2*^+/−^ vs. WT, and 4954 transcripts in *TscD*^+/−^ vs. WT), the expression of 180, 79, and 174 transcripts was significantly reversed by rapamycin treatment in *Tsc1*^+/−^, *Tsc2*^+/−^, and *TscD*^+/−^ mice, respectively (two-way ANOVA, *p* < 0.05; Fig. 4A). The pathway enrichment analysis was performed with canonical pathway modeling in MetaCore to elucidate signal transduction pathways that are related to DETs between mutant and WT mice and are reversed by rapamycin. These DETs were enriched in seven networks, five networks, and six networks in *Tsc1*^+/−^, *Tsc2*^+/−^, and *TscD*^+/−^ mice, respectively, and “signal transducer and activator of transcription 3 (STAT3), interferon regulatory factor 1 (IRF1), IRF4, interleukin-2R (IL-2R) α chain, and interferon-γ (IFN-γ)” was the common network in all mutant mice (Fig. 4A). Most DETs in the network were regulated by STAT3, which was downstream of IRF1, IRF4, IL-2R α chain, and IFN-γ, as well as STAT4 (Fig. 5). The network was initiated from PDZ and LIM domain protein 2 (PDLIM2), which was the only gene that overlapped among DETs in all mutant mice. The gene expression of *Pdlim2* was higher in mutant mice than in WT mice and reduced by rapamycin treatment (Fig. 4B).

## Discussion

Autism spectrum disorder is a polygenic and genetically heterogeneous disorder for which many genes and genetic loci have been identified as causal factors in human genetic studies. The contribution of each gene or genetic locus to ASD is low (up to a few %), indicating that no genetic model covers all or most all ASD cases. The mTOR signaling pathway is a convergent pathway that is disturbed by genetic variations in the *TSC1* and *TSC2* (TSC), *PTEN* (Bannayan-Riley-Ruvalcaba syndrome, Lhermite-Duclos syndrome, and Cowden syndrome), *FMRP* (fragile X syndrome), and *NF1* (neurofibromatosis type 1) genes. All of these disorders are associated with ASD, which together account for approximately 3–5% of overall ASD cases [12]. Thus, *Tsc1/2* heterogeneous knockout mice are considered models for at least 3–5% of ASD cases.

Tuberous sclerosis complex manifests with such neurological symptoms as epilepsy, intellectual disability, and syndromic ASD, the symptoms of which do not differ from non-syndromic ASD [37]. Therefore, TSC is a disease model of the pathogenesis of ASD. Tuberous sclerosis complex is considered to be caused by pathogenic germline mutations (e.g., single nucleotide variations, deletions, insertions, and copy number variations) in either the *TSC1* or *TSC2* gene [38–40]. These mutations were reported to be mostly structural mutations that induce missense (7–33%) and frameshift (24–29%) mutations and affect splice sites (13–40%), whereas 18–28% are nonsense mutations. A total of 9–15% of TSC patients were found to have no identifiable mutation, and genotype–phenotype causal relationships have not yet been elucidated [39–41]. Furthermore, somatic *TSC1* mutations were identified in lesions from patients with a pathogenic germline mutation of *TSC2* [21, 27, 28], suggesting that *TSC1* and *TSC2* mutations may work in concert to elicit phenotypic differences among TSC patients. The present study was performed with *TscD*^+/−^ mice as a model of TSC patients with both *TSC1* and *TSC2* mutations.

*Tsc1*^+/−^ mice were previously reported to exhibit lower active interaction in the social interaction test [34, 36]. *Tsc2*^+/−^ mice and Ekar (*Tsc2*^+/−^) rats were also reported to exhibit lower active interaction in the social interaction test [36, 42, 43] and impairments in social behavior in the social novelty session of the three-chambered test [42], but they did not exhibit abnormalities in the social approach session of the three-chambered test [42, 44]. The present results indicated that heterozygous *Tsc2* mutation-induced social impairment was slightly more severe than the heterozygous *Tsc1* mutation. *TscD*^+/−^ mice exhibited impairments in social behaviors that were similar to *Tsc2*^+/−^ mice and were associated with the mTOR pathway. Tsc1 and Tsc2 proteins form a heterodimeric complex that constitutively inactivates mTORC1 through inhibition of the small guanosine triphosphatase (GTPase) protein Rheb. Tsc2 possesses the GTPase-activating protein (GAP) domain for Rheb in the C terminus, and complex formation with Tsc1 stabilizes Tsc2 and enhances its GAP activity [16]. *TscD*^+/−^ mice were expected to exhibit additive or synergistic phenotypes of both *Tsc1*^+/−^ and *Tsc2*^+/−^ mice. Indeed, the number of astrocytes in the hippocampus was reported to be higher in *TscD*^+/−^ mice than in *Tsc1*^+/−^ and *Tsc2*^+/−^ mice [30]. *Tsc1*^f/−^; *Tsc2*^f/−^; hGFAP-Cre mice have a shorter lifespan than mice with mutations of any of these genes alone [31]. Additionally, glial-fibrillary acidic protein (GFAP)-positive cell-specific *Tsc2* conditional knockout (*Tsc2*^GFAP1^) mice exhibited a high frequency of seizures and shorter life span than *Tsc1*^GFAP1^ conditional knockout mice [45]. Taken together with the present results, *Tsc2* rather than *Tsc1* appears to be dominant for causing neurological symptoms, including impairments in social behaviors that are caused by heterozygous *Tsc1* and/or *Tsc2* mutations, and impairments in social behaviors in *TscD*^+/−^ mice would be partially mediated through different mechanisms from astrocytic abnormalities in an mTOR-dependent manner.

Unlike humans, which have marked individual differences, inbred mouse strains on homogeneous genetic backgrounds can be analyzed with much smaller sample sizes than humans. Indeed, a microarray analysis with 6–8 mice or 57 humans reported that standard deviations of the log (sample/reference) were approximately 0.3 and 0.8, respectively [46]. The Agilent microarray has superior detection compared with other microarray platforms [47]. Our transcriptome data that were based on Agilent microarrays showed that standard deviations of log (mutant/WT) in *Tsc1*^+/−^, *Tsc2*^+/−^, and *TscD*^+/−^ mice were as low as 0.158, 0.165, and 0.226, respectively (Fig. 3A). Therefore, because the mice that were used in the present study were on a homogeneous genetic background and the standard deviation for each mutant group was low, the transcriptome analysis in which four mice were analyzed for each genotype and treatment group was considered to have the necessary power to detect differences between groups. Brain structures are left–right asymmetrical and have different functions that are controlled by each hemisphere [48], and these asymmetries of brain structures and functions have been reported to be broken in ASD [49–51]. Left hemisphere impairments and microstructural abnormalities were found in high-functioning ASD [52, 53], whereas the right hemisphere failed to respond to temporal novelty in ASD [54]. Social deficits in ASD have been reported to be associated with gamma-band electroencephalographic asymmetry in the right hemisphere [55], and social processing is right hemisphere-dominant in vertebrates [56, 57]. Unknown are the precise brain regions and cellular subtypes that are responsible for social deficits in ASD that are associated with TSC. Therefore, whole transcriptome analysis was performed using the right brain hemisphere rather than the left hemisphere and specific brain regions in the present study.

Over 1,000 genes with alterations of gene expressions in each mutant mice compared with WT mice were identified in the study. The magnitude of changes in gene expression in *TscD*^+/−^ mice was higher than in *Tsc1*^+/−^ and *Tsc2*^+/−^ mice. The number of transcripts whose expression was significantly different from WT mice was also higher in *TscD*^+/−^ mice than in *Tsc1*^+/−^ and *Tsc2*^+/−^ mice. Our results indicated that unlike social behaviors, gene expression in the brain in *TscD*^+/−^ mice was affected by synergistic effects of heterozygous *Tsc1* and *Tsc2* mutations. In the present study, few genes in the mTOR1 signaling pathway had alterations of expression in addition to the *Tsc1* and *Tsc2* genes. Similarly, genes in the mTOR signaling pathway were not enriched in the gene expression analysis of cortical tubers from TSC patients, suggesting that transcription does not necessarily feedback from changes in translation, whereas mTOR1 signaling regulates translation via ribosomal protein S6 kinase, polypeptide 1 (S6K1), and eukaryotic translation initiation factor 4E binding protein 1 (4E-BP1) [21, 58]. Transgenic mice in which eukaryotic translation initiation factor 4E (*Eif4e*) gene expression was driven by the β-actin promoter exhibited autistic-like behaviors and a ~ 30% increase in cap-dependent translation, but the enhanced translation was limited to only a part of proteins [59]. Our results indicated that all mutant mice exhibited impairments in social behaviors despite large differences in the number of genes with alterations of gene expression between mutant and WT mice, suggesting that impairments in social behaviors that were caused by *Tsc1* and *Tsc2* mutations would be attributable to changes in specific molecular pathways and not a general increase in transcription or translation.

The hierarchical clustering analysis indicated that gene expression patterns in overlapping genes with alterations of expression between mutant and WT mice were similar in *Tsc2*^+/−^ and *TscD*^+/−^ mice, which corresponded to the severity of impairments in social behavior in mutant mice. Furthermore, genes whose expression was different between *Tsc1*^+/−^ and *Tsc2*^+/−^ mice were mostly enriched in the group of genes that were altered in *Tsc2*^+/−^ and *TscD*^+/−^ mice but not in *Tsc1*^+/−^ mice compared with WT mice, also indicating the similarity of changes in gene expression between *Tsc2*^+/−^ and *TscD*^+/−^ mice (Additional file 2: Fig. S2A). The GO analysis of overlapping genes showed that they were enriched in cancer/neoplasm- and immunity/inflammation-related processes. These cancer/neoplasm- and immunity/inflammation-related processes showed the up- and downregulation of genes that were altered in *Tsc2*^+/−^ and *TscD*^+/−^ mice (Additional file 2: Fig. S2B), respectively, indicating that the severity of symptoms that are related to cancer/neoplasm and immunity/inflammation would be different between TSC patients with underlying *TSC1* and *TSC2* mutations.

STAT3 is phosphorylated by mTOR1 and activated in *TSC2*-deficient cells, which are inhibited by rapamycin [60–62]. The expression of inflammatory gene markers, including IL-1β, IL-6, and Toll-like receptors, and phosphorylated STAT3 increased in brain lesions in TSC patients [63–66]. These previous studies support our findings that the “STAT3, IRF1, IRF4, IL-2R α chain, IFN-γ” pathway network was associated with impairments in social behaviors in mutant mice and suggest that suppressing inflammatory responses in TSC patients may improve social deficits. This pathway network is initiated by *Pdlim2*, which is ubiquitously expressed throughout the body of mice but especially in the genitourinary, nervous, and visceral organ systems. *Pdlim2* encodes an E3 ubiquitin ligase and ubiquitinates STAT3 and STAT4 to lead to their degradation [67, 68]. Pdlim2 is reported to be associated with tumor suppression [69–71] and tumorigenesis [72, 73], but its function in the nervous system remains unknown.

## Limitations

The “STAT3, IRF1, IRF4, IL-2R α chain, IFN-γ” pathway network initiated from PDLIM2 and STAT4 was associated with impairments in social behaviors in *Tsc1*^+/−^, *Tsc2*^+/−^, and *TscD*^+/−^ mice, but the regulatory mechanisms of the mTOR pathway and social behaviors by the pathway network were not elucidated in the present study. It is also unclear whether the pathway network also plays a critical role in impaired social behaviors in autism spectrum disorders not caused by *Tsc1* and *Tsc2* mutations.

## Conclusions

In the present study, detailed analyses of social behaviors and transcriptomes in *Tsc1*^+/−^, *Tsc2*^+/−^, and *TscD*^+/−^ mice revealed the genetic pathology of impairments in social behaviors. Mutations in the *TSC2* gene are likely to have dominant effects on social behaviors rather than *TSC1* gene if these mutations have similar potency. However, rapamycin has therapeutic effects on impairments in social behaviors caused by the mutations in both genes. It is also suggested that social behavioral abnormalities in patients with mutations in both the *TSC1* and *TSC2* genes are similar to those in patients with mutations in the *TSC2* gene. The Pdlim2-mediated signaling pathway is suggested to be associated with altered brain function and impaired social behaviors due to mutations in the *TSC1* and *TSC2* genes. The present findings further our understanding of genotype–phenotype relationships and may contribute to the development of medications that can ameliorate social deficits in ASD.

## Supplementary Information

**Additional file 1**. **Fig. S1.** Anxiety and sensory behaviors in *Tsc1*^+/−^, *Tsc2*^+/−^, and *TscD*^+/−^ mice.

**Additional file 2**. **Fig. S2.** Differences in changes in gene expression between *Tsc1*^+/−^ and *Tsc2*^+/−^ mice.

**Additional file 3**. **Table S1.** GO enrichment analysis of significant DETs in the brain in *Tsc1*^+/−^, *Tsc2*^+/−^, and *TscD*^+/−^ mice.

**Additional file 4**. **Table S2.** Canonical model pathways commonly enriched in the brain in *Tsc1*^+/−^, *Tsc2*^+/−^, and *TscD*^+/−^ mice.

## Data Availability

The datasets used and/or analyzed during the current study are available from the corresponding author on reasonable request.

## Abbreviations

4E-BP1: 4E binding protein 1
ANOVA: Analysis of variance
ASD: Autism spectrum disorder
DETs: Differentially expressed transcripts
FDR: False discovery rate
GAP: GTPase-activating protein
GFAP: Glial-fibrillary acidic protein
GO: Gene ontology
GTPase: Guanosine triphosphatase
HSD: Honestly significant difference
IFN-γ: Interferon-γ
IL-2R: Interleukin-2R
IRF1: Interferon regulatory factor 1
LOH: Loss of heterozygosity
mTORC1: Mechanistic target of rapamycin complex 1
PDLIM2: PDZ and LIM domain protein 2
S6K1: S6 kinase, polypeptide 1
STAT3: Signal transducer and activator of transcription 3
TSC: Tuberous sclerosis complex
*TSC1*: TSC complex subunit 1
*Tsc1*^+/−^: *Tsc1* Heterozygous knockout
*Tsc2*^+/−^: *Tsc2* Heterozygous knockout
*TscD*^+/−^: *Tsc1* And *Tsc2* double heterozygous mutant
WT: Wild type
