# Supplementary material for: Tsc2 mutation rather than Tsc1 mutation dominantly causes a social deficit in a mouse model of tuberous sclerosis complex
Source: Hum Genomics. 2023 Feb 2;17:4. doi: 10.1186/s40246-023-00450-2 (PMC9893559; doi:10.1186/s40246-023-00450-2)
Supplement: Supplementary file 1 — Additional file 1. Fig. S1. Anxiety and sensory behaviors in Tsc1+/−, Tsc2+/−, and TscD+/− mice. [file 40246_2023_450_MOESM1_ESM.pdf]

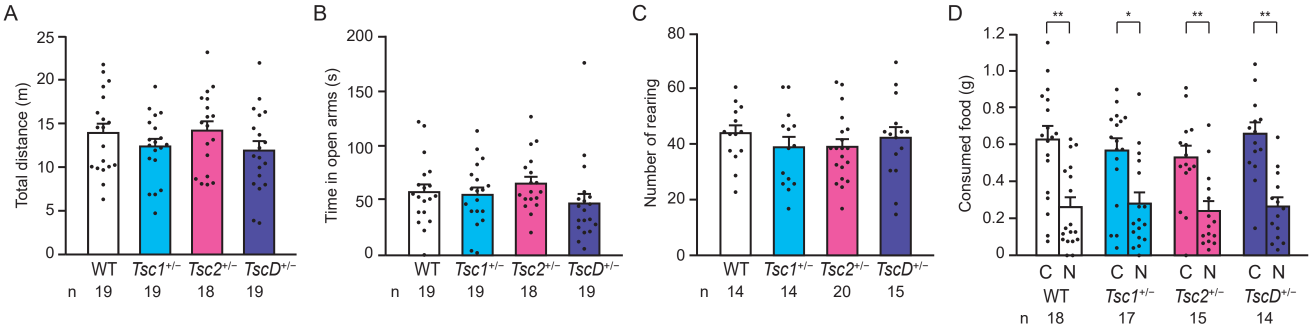

Fig. S1. Anxiety and sensory behaviors in *Tsc1*$^{+/-}$, *Tsc2*$^{+/-}$, and *TscD*$^{+/-}$ mice.

(**A, B**) Total distance travelled (**A**) and total time spent on the open arms (**B**) in the elevated plus-maze test. (**C**) Total time spent in rearing behavior in the self-grooming test. (**D**) Consumption of preferred cued food (C) and non-cued food (N) in the social transmission of food preference test. *$p < 0.05$, **$p < 0.01$, one-way ANOVA followed Tukey HSD test (**A-C**) and paired *t*-test (**D**). Each bar indicates the mean ± SEM.
