# Supplementary material for: Tsc2 mutation rather than Tsc1 mutation dominantly causes a social deficit in a mouse model of tuberous sclerosis complex
Source: Hum Genomics. 2023 Feb 2;17:4. doi: 10.1186/s40246-023-00450-2 (PMC9893559; doi:10.1186/s40246-023-00450-2)
Supplement: Supplementary file 2 — Additional file 2. Fig. S2. Differences in changes in gene expression between Tsc1+/− and Tsc2+/− mice. [file 40246_2023_450_MOESM2_ESM.pdf]

A

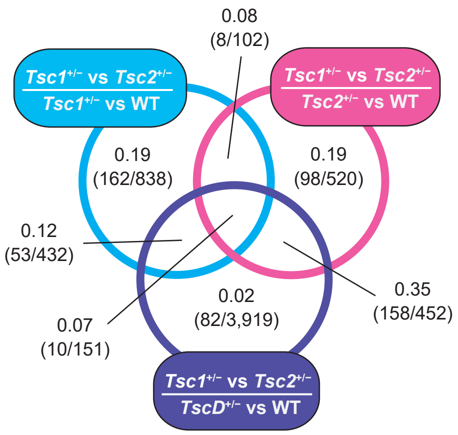

B

Rank	Map Folders (*Tsc1*$^{+/-}$ U *Tsc2*$^{+/-}$ U *TscD*$^{+/-}$)
1	Lung cancer
2	Systemic Lupus Erythematosus
3	Asthma
4	Melanoma
5	Depression
6	Inflamatory response
7	Immuno system response
8	Colorectal Neoplasms
9	Stomach Neoplasms
10	Neurofibromatoses
11	Cell cycle and its regulation
12	Pancreatic Neoplasms
13	Carcinoma, Hepatocellular
14	Cell differentiation
15	Tissue remodelong and wound repair
16	Dermatitis, Allergic Cntact
17	Ovarian cancer
18	DNA-damage response
19	Multiple myeloma
20	Hematology

Rank	Map Folders (*Tsc1*$^{+/-}$ vs *Tsc2*$^{+/-}$)
1	Pancreatic Neoplasms
2	DNA-damage response
3	Colorectal Neoplasms
4	Lung cancer
5	Cell cycle and its regulation
6	Ovarian cancer
7	Immuno system response
8	Asthma
9	Stomach Neoplasms
10	Prostatic Neoplasms
11	Systemic Lupus Erythematosus
12	Inflamatory response
13	Carcinoma, Hepatocellular
14	Multiple myeloma
15	Transcription regulation
16	Melanoma
17	Breast Neoplasms
18	Neurofibromatoses
19	Dermatitis, Allergic Cntact
20	Stem cells

Fig. S2. Differences in changes in gene expression between *Tsc1*$^{+/-}$ and *Tsc2*$^{+/-}$ mice.

(**A**) Venn diagram of ratios of DETs between *Tsc1*$^{+/-}$ and *Tsc2*$^{+/-}$ mice in three DETs between mutant and WT mice. (**B**) Top 20 rank Map folders enriched for DETs between mutant and WT mice and between *Tsc1*$^{+/-}$ and *Tsc2*$^{+/-}$ mice. The blue and red folders indicate cancer/neoplasm and immunity/inflammation, respectively.
