# Supplementary material for: Tsc2 mutation rather than Tsc1 mutation dominantly causes a social deficit in a mouse model of tuberous sclerosis complex
Source: Hum Genomics. 2023 Feb 2;17:4. doi: 10.1186/s40246-023-00450-2 (PMC9893559; doi:10.1186/s40246-023-00450-2)
Supplement: Supplementary file 3 — Additional file 3. Table S1. GO enrichment analysis of significant DETs in the brain in Tsc1+/−, Tsc2+/−, and TscD+/− mice. [file 40246_2023_450_MOESM3_ESM.pdf]

Table S1. GO enrichment analysis of significant DETs in the brain in *Tsc1*$^{+/-}$, *Tsc2*$^{+/-}$, and *TscD*$^{+/-}$ mice.

Rank #	Biological Process	p-Value	FDR	Ratio
1	cellular component organization or biogenesis	8.56E-11	3.44E-07	320/7183
2	regulation of cation channel activity	9.22E-11	3.44E-07	28/198
3	nervous system development	2.08E-10	4.18E-07	173/3307
4	cellular component organization	2.24E-10	4.18E-07	309/6929
5	regulation of cytosolic calcium ion concentration	1.74E-09	2.26E-06	39/399
6	response to abiotic stimulus	1.81E-09	2.26E-06	110/1870
7	cellular localization	3.88E-09	4.14E-06	144/2710
8	response to endogenous stimulus	5.29E-09	4.49E-06	133/2454
9	negative regulation of gonadotropin secretion	5.41E-09	4.49E-06	9/20
10	central nervous system development	7.45E-09	5.14E-06	92/1505
11	regulation of ion transmembrane transporter activity	8.00E-09	5.14E-06	31/287
12	regulation of cation transmembrane transport	8.26E-09	5.14E-06	37/387
13	regulation of receptor activity	1.00E-08	5.76E-06	25/198
14	establishment of localization in cell	1.10E-08	5.86E-06	116/2074
15	regulation of transmembrane transporter activity	1.53E-08	7.63E-06	31/295
16	regulation of transporter activity	2.54E-08	1.12E-05	32/318
17	head development	2.60E-08	1.12E-05	78/1231
18	system development	2.70E-08	1.12E-05	270/6129
19	regulation of cell communication	3.34E-08	1.22E-05	195/4112
20	phospholipase C-activating G-protein coupled receptor signaling pathway	3.53E-08	1.22E-05	22/167

Top 20 processes from GO Process Networks in MetaCore for 1,119 transcripts. FDR, false discovery rate.
