# Supplementary material for: Tsc2 mutation rather than Tsc1 mutation dominantly causes a social deficit in a mouse model of tuberous sclerosis complex
Source: Hum Genomics. 2023 Feb 2;17:4. doi: 10.1186/s40246-023-00450-2 (PMC9893559; doi:10.1186/s40246-023-00450-2)
Supplement: Supplementary file 4 — Additional file 4. Table S2. Canonical model pathways commonly enriched in the brain in Tsc1+/−, Tsc2+/−, and TscD+/− mice. [file 40246_2023_450_MOESM4_ESM.pdf]

Table S2. Canonical model pathways commonly enriched in the brain in *Tsc1*$^{+/-}$, *Tsc2*$^{+/-}$, and *TscD*$^{+/-}$ mice.

No	Key network objects	GO Processes	Total nodes	Seed nodes	p-Value	zScore	gScore
1	c-Myc, Alpha-catenin, Cyclin D1, K-RAS, SIAT8B	cellular response to indole-3-methanol (5.8%), response to indole-3-methanol (5.8%), canonical Wnt signaling pathway involved in negative regulation of apoptotic process (4.3%), regulation of cell death (29.0%), cellular response to alcohol (7.2%)	75	66	4.61E-206	285.82	285.82
2	MTND6, Huntingtin, CREB1, CBP, PICK1	cellular response to hepatocyte growth factor stimulus (7.1%), response to hepatocyte growth factor (7.1%), macromolecule modification (42.9%), positive regulation of non-motile cilium assembly (4.8%), positive regulation of hormone secretion (9.5%)	45	42	5.41E-132	234.81	234.81
3	STAT3, IRF4, IRF1, IL-2R alpha chain, IFN-gamma	cytokine-mediated signaling pathway (28.8%), response to cytokine (33.9%), cellular response to cytokine stimulus (30.5%), phosphorylation (33.9%), JAK-STAT cascade involved in growth hormone signaling pathway (8.5%)	62	44	1.63E-127	209.53	209.53
4	CREB1, Elk-1, c-Fos, c-Jun, PKA -cat (cAMP-dependent)	protein phosphorylation (34.9%), phosphorylation (38.4%), response to organonitrogen compound (34.9%), response to peptide (25.6%), response to nitrogen compound (34.9%)	97	54	3.91E-149	207.71	207.71
5	PPCKM, Insulin processed, Norepinephrine extracellular region, IGF-1, Insulin	response to organonitrogen compound (44.2%), response to nitrogen compound (45.2%), cellular response to peptide (28.8%), cellular response to nitrogen compound (36.5%), cellular response to peptide hormone stimulus (27.9%)	126	53	9.18E-138	179.84	179.84
6	GSTM5, Sirtuin1, AMPK beta subunit, AMP cytoplasm, FOXO3A	cellular response to oxygen levels (26.5%), positive regulation of gluconeogenesis (14.7%), carbohydrate homeostasis (26.5%), glucose homeostasis (26.5%), regulation of fatty acid metabolic process (20.6%)	36	24	3.69E-68	149.96	149.96
7	Elk-1, c-Jun, c-Fos, Galpha(i)-specific peptide GPCRs, Galanin	regulation of Golgi inheritance (13.9%), regulation of early endosome to late endosome transport (16.7%), regulation of cytoplasmic transport (16.7%), trachea formation (13.9%), regulation of Golgi organization (13.9%)	37	24	1.05E-67	147.92	147.92

Canonical pathway modeling analysis was conducted with MetaCore for 148 transcripts that overlapped among DETs between "*Tsc1*$^{+/-}$ vs. WT," "*Tsc2*$^{+/-}$ vs. WT," and "*TscD*$^{+/-}$ vs. WT." $p < 0.05$, zScore > 100.
